# Comprehensive Pediatric Health Risk Stratification Using an AI-Driven Framework in Children Aged 2 to 8 Years: Design and Validation Study

**DOI:** 10.2196/80163

**Published:** 2026-01-26

**Authors:** Zhihe Mao, Jundan Chen

**Affiliations:** 1 School of Physical Education, Hunan University of Arts and Science, 3150 Dongting Road, Changde, Hunan, 415000, China, 86 18832584414, 86 88547123

**Keywords:** pediatric health, artificial intelligence, AI, risk stratification, ensemble learning, framework design, multisource data fusion, predictive modeling

## Abstract

**Background:**

Early life health risks can shape long-term morbidity trajectories, yet prevailing pediatric risk assessment paradigms are often fragmented and insufficiently capable of integrating heterogeneous data streams into actionable, individualized profiles.

**Objective:**

This study aimed to design, implement, and validate an artificial intelligence–driven framework that fuses multimodal pediatric data and leverages advanced natural language processing and ensemble learning to improve early, accurate stratification of key pediatric health risks.

**Methods:**

A retrospective dataset of over 40,000 pediatric participants aged 2‐8 years was used to train and evaluate the framework. Data were split into training, validation, and test sets (70%, 15%, and 15%, respectively) with a temporally mindful partitioning strategy to approximate prospective evaluation. Baseline comparators included traditional statistical and machine learning models, and the statistical significance of area under the receiver operating characteristic curve (AUC-ROC) differences was assessed using the DeLong test.

**Results:**

The proposed Bidirectional Encoder Representations From Transformers–based model achieved an AUC-ROC of 0.85 (95% CI 0.82‐0.88), sensitivity of 0.78, specificity of 0.80, and *F*_1_-score of 0.75 on the test set, outperforming multiple baseline models. In an additional manual comparison evaluation, automated and expert assessments aligned with 78% accuracy (78/100), and most discrepancies arose in “equivalent” cases.

**Conclusions:**

This study provides a validated, artificial intelligence–driven, multimodal pediatric health risk stratification framework that translates heterogeneous child health data into clinically actionable risk profiles, demonstrating strong discriminative performance and meaningful agreement with expert assessment. The framework supports proactive, individualized pediatric care and offers a scalable foundation for further validation across broader populations and longitudinal follow-up.

## Introduction

The assessment and early identification of health risks in children represent a critical area of research with profound implications for individual lifelong well-being and public health strategies. Establishing healthy developmental trajectories during childhood is fundamental, as health conditions and risk exposures in early life can significantly influence adult health outcomes and susceptibility to chronic diseases [1,2]. Traditional pediatric health surveillance often relies on periodic checkups, which may not fully capture the dynamic and multifaceted nature of health risks influenced by a complex interplay of genetic, environmental, and lifestyle factors [3,4]. Therefore, developing advanced, data-driven methodologies, such as those explored in recent research on artificial intelligence (AI) and machine learning (ML) for tasks like toxicity prediction and broader research applications, to proactively identify and stratify these risks is of paramount importance. Such systems, potentially incorporating guardian-interactive elements, can empower health care providers and families with actionable insights for timely preventive interventions, ultimately contributing to a healthier future generation and optimizing health care resource allocation [5]. Global health initiatives increasingly emphasize the importance of leveraging innovative technologies, including advanced AI models, such as large language models adapted for specific domains [6], to enhance child health and well-being. The potential for deep learning to enhance diagnostic and prognostic capabilities in areas like child psychiatry further underscores this trend [7].

The rapid popularization of internet-based medical services, including telehealth, mobile health (mHealth) apps, and online consultation platforms, has significantly reshaped health care delivery and accessibility worldwide [8]. This digital transformation has not only provided patients and caregivers with more convenient access to medical advice and health information but also led to the generation of vast amounts of digital health data [9,10]. While much of the initial adoption has been observed in adult care, the principles of leveraging digital platforms for health monitoring, data collection, and remote consultation, often supported by automated systems for tasks like information synthesis [11], are increasingly recognized as valuable in pediatric care. The experience gained from the broader implementation of digital health solutions, including aspects of data security, interoperability, and user engagement, offers important lessons for developing effective and safe digital health tools specifically for children [12,13]. The expanding digital health infrastructure provides a rich ecosystem for collecting diverse health-related data that, if properly managed and ethically used with robust frameworks, can be invaluable for comprehensive risk assessment in pediatric populations [14,15]. Task-oriented dialogue systems, for instance, show potential for structured data gathering in such contexts. Despite progress in pediatric health surveillance, significant clinical voids persist, particularly in the settings of primary care and population health screening. Current risk assessment paradigms are often fragmented, struggling to effectively integrate heterogeneous data streams, such as structured electronic health record (EHR) data, unstructured clinical notes, parental reports, and real-time wearable sensor data, into a single, cohesive risk profile. This leads to a reactive, rather than proactive, approach to care, where risks are often identified only after they have manifested. This research addresses this gap by proposing a framework designed for the following specific end users: (1) pediatricians in primary care, who can use the system for point-of-care decision support; (2) public health officials, who can leverage population-level risk distributions for resource allocation and strategic planning; and (3) clinical care coordinators, who can manage and monitor high-risk pediatric cohorts more effectively.

The increasing availability of EHR data, administrative data, patient-generated health data, and other health-related information sources has fueled the application of health care big data analytics to improve service quality, operational efficiency, and patient outcomes [16-18]. Effective utilization of these data, supported by primers on data handling and the underlying principles of models like Bidirectional Encoder Representations From Transformers (BERT), can support evidence-based clinical decision-making, enhance population health management, facilitate epidemiological surveillance, and enable the development of personalized health care interventions [19]. To enhance clinical utility and facilitate shared decision-making, it is imperative that the reasons behind a child’s risk stratification are transparent and interpretable. Shapley additive explanations (SHAP) are used due to their strong theoretical guarantee of consistency and local accuracy, which can provide a unified and reliable framework for model interpretation [20]. For a given prediction, SHAP assigns an importance value (SHAP value) to each input feature, and it is crucial for unlocking its full potential in transforming health care delivery, research, and policy.

AI algorithms can analyze complex, multidimensional datasets to identify subtle patterns, predict the likelihood of various health conditions, and stratify risk earlier and more accurately than traditional statistical methods [20,21]. Researchers have explored various AI-driven systems for health applications. For example, some systems focus on the early detection of developmental disorders using behavioral or imaging data, while others aim to predict adverse outcomes in neonatal intensive care units or identify children at risk for conditions like asthma, obesity, and mental health issues, sometimes using specific BERT-based approaches for analyzing medical records or predicting conditions like Alzheimer disease (AD-BERT), which, while not pediatric, demonstrates domain-specific adaptation [22]. Common approaches in developing such systems involve the curation of large, representative datasets; rigorous data preprocessing and feature engineering; the application of appropriate ML algorithms; and robust model validation using independent test sets and ideally prospective clinical evaluation [23]. For instance, deep learning models have shown promise in analyzing medical images for pediatric conditions, while ensemble methods and sophisticated scoring mechanisms like BERTScore for evaluating text generation quality [24] (analogous to assessing the quality of AI-generated health summaries) are often used to improve the robustness and accuracy of predictive models based on structured EHR data or textual information [25]. The primary contribution of our framework lies in its advanced natural language processing (NLP) capabilities, which distinguish it from prior AI systems in pediatric care that have largely relied on traditional NLP techniques. Methodologies, such as keyword matching, bag-of-words, and term frequency-inverse document frequency models, while useful, are fundamentally limited in their ability to interpret the complex and nuanced narratives found in clinical texts.

Therefore, this study aimed to design, implement, and validate a comprehensive, AI-driven framework for pediatric health risk stratification. We hypothesize that by integrating multimodal data, including EHRs, parental questionnaires, and wearable sensor data, and leveraging advanced NLP and ensemble learning models, our system can identify and stratify key pediatric health risks (eg, obesity and developmental delay) with greater accuracy and at an earlier stage than traditional assessment methods, thereby providing robust support for clinical decision-making and pre-emptive intervention. A comparison of pediatric risk stratification frameworks is provided in Table 1.

**Table 1.** Comparison of the selection of pediatric risk stratification frameworks.

Study	Key method	Data modalities used	Clinical deployment status
Smith et al [26]	Logistic regression	EHR^a^ (structured only)	Retrospective validation
Chen et al [27]	Random forest	EHR and parental surveys	Prototype on retrospective data
Our study	Fine-tuned BERT^b^ + ensemble	EHR, surveys, and wearables	Prototype on retrospective data
Lee et al [28]	CNN^c^ on images	Medical imaging	Conceptual framework

a EHR: electronic health record.

b BERT: Bidirectional Encoder Representations From Transformers.

c CNN: convolutional neural network.

## Methods

### Pediatric Health Risk Stratification Framework and Data Foundation

This section addresses the critical, complex, and multifaceted aspects of multimodal data acquisition, encompassing the collection of diverse data types from various sources and the requisite, often intricate, data preparation processes. These preparation stages, including but not limited to data cleaning, normalization, transformation, and feature engineering, are indispensable for effectively fueling the advanced AI models that form the analytical core of this research, ensuring they operate on high-quality, meaningful inputs to generate reliable and actionable insights [29,30]. The overarching goal is to establish robust data and conceptual groundwork upon which sophisticated risk prediction and stratification can be built, ultimately aiming to transform pediatric health care through proactive and personalized interventions. Conceptually, the framework aims to map a complex set of input variables $X_{\text{data }}$ (representing multimodal pediatric data) through a series of transformations and AI modeling of $f_{AI}$ to an interpretable risk stratification outcome $Y_{\text{risk }}$:


(1) $$Y_{\text{risk}}=f_{AI}\left(\text{Preprocessing}\left(X_{\text{data}}\right)\right)$$

This high-level representation underscores the journey from raw data to actionable risk assessment.

### Conceptual Framework for Pediatric Risk Stratification

**Figure 1.**
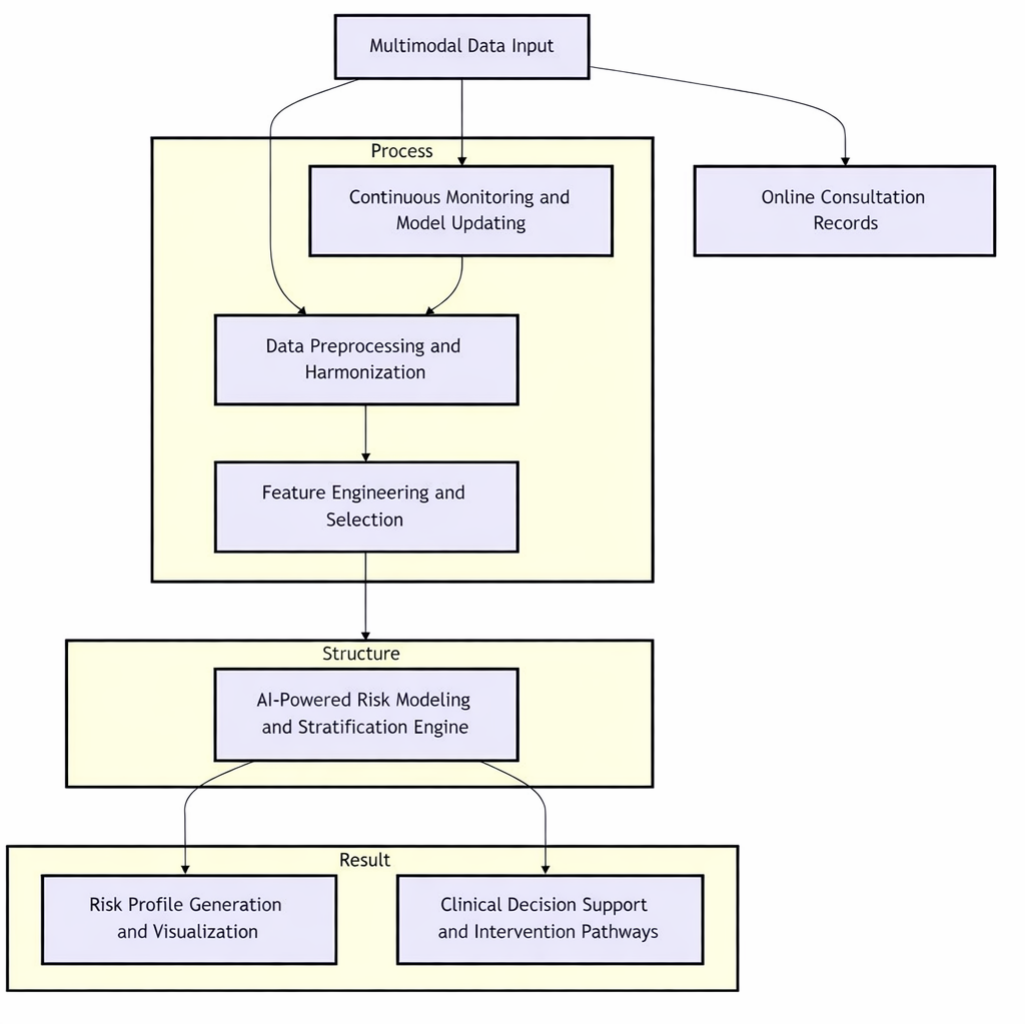
Conceptual diagram of the AI-driven pediatric health risk stratification framework. The image visually represents the modules and their interconnections. AI: artificial intelligence.

The creation of a robust pediatric health risk stratification system requires a meticulously defined conceptual framework that delineates the core components, their interactions, and the overall data flow. As depicted in Figure 1, the proposed AI-driven framework is architected to be modular, scalable, and adaptable to diverse pediatric health contexts. Its primary objective is to convert raw, multisource pediatric data into actionable risk profiles, thereby facilitating proactive and personalized health care interventions.

Commencing with *multimodal data input*, the framework assimilates a wide array of data sources pertinent to child health. Subsequently, the *data preprocessing and harmonization* layer comes into play. Given the heterogeneity and potential quality issues inherent in real-world pediatric data, such as missing values, inconsistencies, and varying formats from different sources, this layer uses data cleaning, normalization, transformation, and integration techniques to create a unified and analysis-ready dataset.

Next is the *feature engineering and selection* module, which is dedicated to extracting meaningful features from the preprocessed data that are highly indicative of pediatric health risks. This process may involve creating composite variables, transforming existing features, or applying dimensionality reduction techniques to optimize the input for AI models. Domain knowledge from pediatric medicine is crucial in guiding this process to ensure clinical relevance.

At the core of the framework lies the *AI-powered risk modeling and stratification engine*. This engine uses advanced ML algorithms, with a particular focus on ensemble learning techniques, as discussed in the Introduction, to construct predictive models for various pediatric health risks. Trained on historical data, these models learn complex patterns and relationships between input features and health outcomes or risk states. The output of this engine is not merely a binary prediction but a nuanced risk stratification, which may encompass risk scores, probability estimates, or the identification of distinct risk phenotypes. The determination of weights for each data modality is a critical aspect of our ensemble model. Rather than assigning predetermined, fixed weights, our *stacking* framework uses a data-driven approach.

The final layers of the framework are devoted to *risk profile generation and visualization* and *clinical decision support and intervention pathways*. The generated risk profiles are designed to be interpretable by health care professionals and understandable by caregivers, often incorporating visual aids and clear explanations. This output is intended to seamlessly integrate with clinical workflows, offering evidence-based insights to support shared decision-making regarding preventive strategies, further assessments, or targeted interventions. *Continuous monitoring* and *model updating* are also integral to the framework, ensuring that the AI models maintain their accuracy and relevance as new information emerges and clinical knowledge evolves [31].

### Identification of Key Pediatric Health Domains and Risk Factors

The primary pediatric health domains identified for this framework, as detailed in Table 2, cover a broad spectrum of child well-being. *Patient safety* focuses on risks, such as adverse drug events and medical errors, typically informed by EHR data and incident reports. *Continuous health monitoring* is crucial, tracking growth parameters and developmental milestones using EHR data and wearable sensor inputs. For children with ongoing conditions, *chronic disease management* addresses factors like glycemic control or asthma exacerbation frequency, primarily using EHR data and home monitoring device information. *Mental health* is a significant domain, evaluating behavioral indicators and emotional well-being via parental, teacher, and direct child assessments. Furthermore, *preventive care* adherence is monitored through risk factors, such as vaccination status and well-child visit compliance, drawing from EHR data and parental questionnaires [32]. The interconnected domains of *nutrition* and *growth* consider risks associated with nutritional intake and growth percentiles, also informed by EHR and parental questionnaire data. Lastly, *physical activity* levels and sedentary behavior duration, identified as critical risk factors, are assessed using wearable sensor data and parental questionnaires.

For each identified health domain, a set of specific, measurable risk factors and associated potential data sources is cataloged. This systematic identification ensures that the subsequent data acquisition and AI modeling efforts are focused and aligned with the goal of comprehensive risk assessment. The dynamic nature of these risk factors across different developmental stages (infancy, early childhood, middle childhood, and adolescence) is also a key consideration in the framework’s design and application.

**Table 2.** Key pediatric health domains, associated risk factors, and potential data sources.

Key pediatric health domains	Associated risk factors	Potential data sources
Patient safety	Adverse drug events and medical errors	EHR^a^ data and incident reports
Health monitoring	Growth parameters and developmental milestones	EHR data and wearable sensor data
Chronic disease management	Glycemic control and asthma exacerbation frequency	EHR data and home monitoring devices
Acute illness severity	Fever severity and respiratory distress indicators	EHR data and parental questionnaire responses
Mental health	Behavioral indicators and emotional well-being	Parental and teacher assessments, and direct child assessments
Preventive care	Vaccination status and well-child visit adherence	EHR data and parental questionnaire responses
Nutrition and growth	Nutritional intake and growth percentiles	EHR data and parental questionnaire responses
Physical activity	Activity levels and sedentary behavior duration	Wearable sensor data and parental questionnaire responses

a EHR: electronic health record.

### Multimodal Data Acquisition and Preparation

The effectiveness of the AI-driven risk stratification framework is fundamentally dependent on the availability of comprehensive, high-quality, multimodal data that accurately reflect the various factors influencing child health. This section details the strategies for acquiring and preparing such data for input into the framework, emphasizing the critical importance of ethical considerations, including informed consent, data privacy, and security, in full compliance with relevant regulations, such as the General Data Protection Regulation (GDPR) and Health Insurance Portability and Accountability Act (HIPAA), adapted for pediatric populations.

The data sources for this framework are inherently multimodal, encompassing a variety of information types. EHRs serve as a primary source, offering longitudinal clinical data that include diagnoses (ICD codes), procedures, medications, laboratory results, growth chart data, and clinical notes. Access to both structured and unstructured EHR data is essential. *Parental and child questionnaires or surveys* are used to capture information often not systematically recorded in EHRs, such as detailed family history, socioeconomic status, lifestyle factors (diet, physical activity, and sleep), environmental exposures, and patient-reported outcomes or symptoms. *Wearable sensors and mHealth data* provide opportunities for continuous, real-world monitoring of physiological parameters (eg, heart rate, activity levels, and sleep patterns) and behavioral data. *School health records* can contribute information on immunizations, health screenings conducted at school, and potentially attendance or behavioral notes relevant to health [33]. *Public health and environmental databases* can offer community-level data on factors like air quality, neighborhood socioeconomic indicators, and access to health care or recreational facilities. *Genomic data*, when applicable and ethically sourced, can provide valuable genetic risk scores or specific genetic markers associated with pediatric conditions.

**Figure 2.**
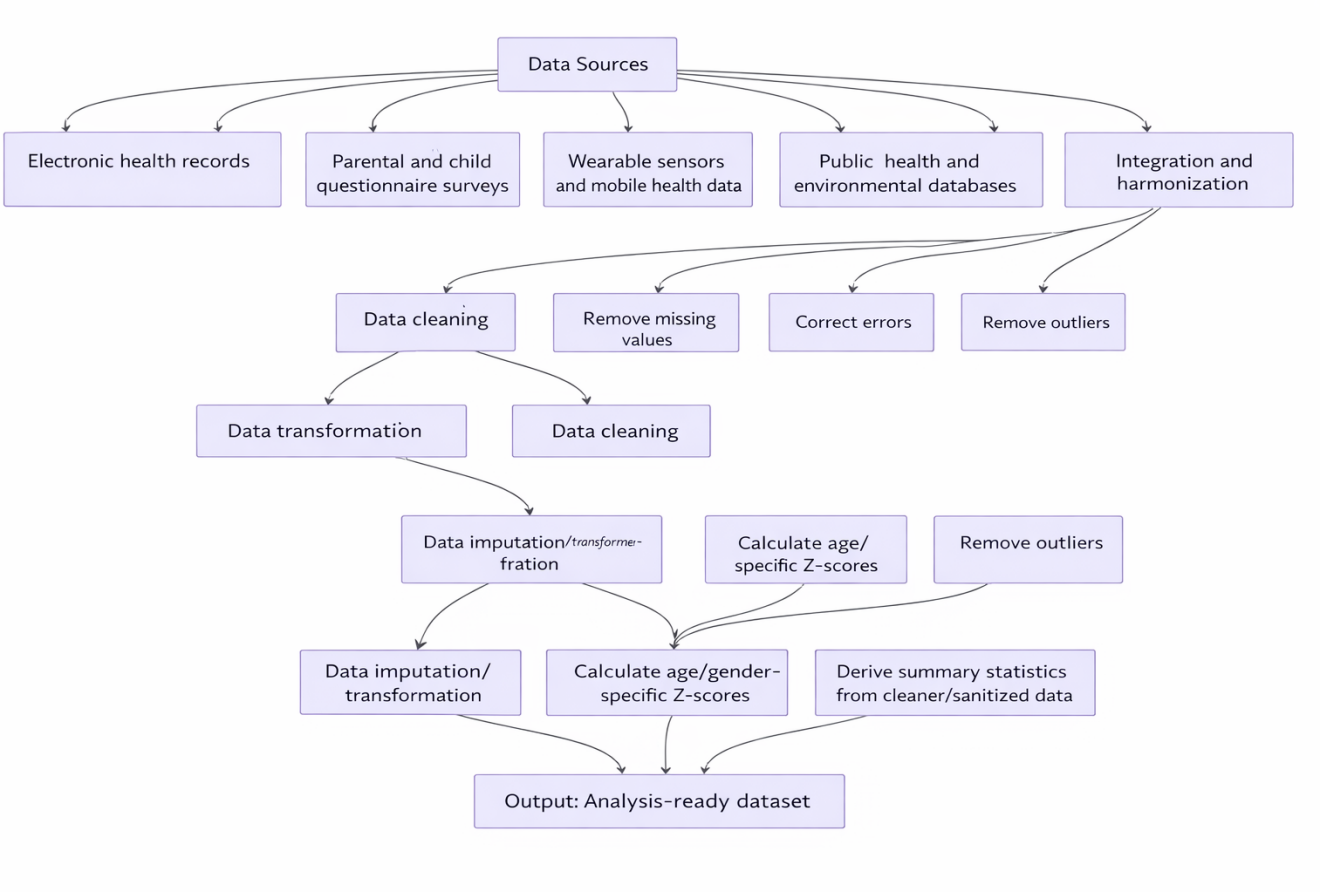
Multimodal data acquisition and preparation pipeline.

The data preparation pipeline, illustrated in Figure 2, involves several key steps to transform raw, multimodal data into an analysis-ready dataset. Initially, data from disparate sources must undergo integration and harmonization. This process includes mapping data elements to common terminologies, resolving inconsistencies, and creating a unified data schema. Data cleaning is then performed to address missing values, correct errors, and remove outliers. Data transformation may be necessary to convert raw data into formats suitable for analysis, such as calculating age-specific *z* scores for growth parameters or deriving summary statistics from time-series sensor data. For instance, if $x_{raw}$ is a raw measurement and $\mu_{age,sex}$ and $\sigma_{age,sex}$ are the age- and sex-specific mean and SD, respectively, from a reference population, the *z* score can be calculated as follows:


$$z=\frac{x_{raw}-\mu_{age,sex}}{\sigma_{age,sex}}$$

This normalization is crucial for enabling comparisons of measurements across different age groups and sexes. Finally, data anonymization or pseudonymization is rigorously applied to protect patient privacy before the data are used for model development. The resulting dataset provides the foundation for the feature engineering and AI modeling stages described in the subsequent part.(2)

### Data Preprocessing and Feature Engineering

The integrity and utility of input data are paramount to the performance and clinical relevance of any AI model, particularly in the pediatric domain, where data can be sparse, longitudinal, and highly variable due to growth and development. Pediatric health data, often derived from multiple heterogeneous sources, such as EHRs, parental questionnaires, school health notes, and wearable sensors, typically present unique challenges, including systematically missing values (eg, developmental assessments not performed at certain ages), measurement noise (eg, variability in home-based measurements), and the presence of irrelevant or redundant information that can obscure true risk signals. Therefore, rigorous data preprocessing and thoughtful, domain-informed feature engineering are indispensable steps to prepare a high-quality dataset for subsequent AI modeling.

The initial phase of preprocessing involves comprehensive data cleaning. Missing data represent a pervasive issue in longitudinal pediatric health care datasets. For instance, growth parameters or developmental screening results might be missing for specific well-child visits. While simple imputation techniques like mean, median, and mode imputation can be applied for variables with a low percentage of missingness and random patterns, they often fail to capture the underlying data structure in pediatric cohorts. More sophisticated methods are typically required, such as k-nearest neighbors imputation, which identifies the k most similar pediatric cases (based on a suite of other observed features) and imputes the missing value for a feature $X_{j}$ using a weighted average or majority vote from the neighbors [34]. Alternatively, model-based imputation, using algorithms like multivariate imputation by chained equations (MICE), can be used. MICE iteratively models each variable with missing values as a function of other variables in the dataset, cycling through variables until convergence is achieved. For a set of variables $X_{1},\ldots ,X_{p}$, MICE specifies a conditional model $P\left(X_{j}|X_{-j},\phi_{j}\right)$ for each $X_{j}$ and iteratively samples from these conditional distributions.

Outlier detection and appropriate treatment are also critical in pediatric data, where extreme values might represent genuine clinical concern or measurement error. Statistical methods, such as the *z* score and IQR, are used. For a data point $x_{i}$ in a feature distribution, its *z* score is calculated as $z_{i}=\left(x_{i}-\mu \right)/\sigma$, where μ is the mean and σ is the SD. Data points with $\left|z_{i}\right|>\theta_{outlier}$ may be flagged. The IQR method defines outliers as points falling below $Q_{1}-1.5\times IQR$ or above $Q_{3}+1.5\times IQR$, where $Q_{1}$ and $Q_{3}$ are the first and third quartiles, respectively, and $IQR=Q_{3}-$$Q_{1}$. Decisions on handling outliers (removal, transformation, or winsorization) are made cautiously, considering the potential clinical significance of extreme values in child health.

Feature engineering in the pediatric context is a highly domain-driven process focused on creating new, informative features from raw data to capture developmental trajectories, critical exposure periods, and clinically relevant interactions. This enhances model performance and interpretability. In child health, this includes deriving age- and sex-adjusted *z* scores for growth parameters, such as height, weight, BMI, and head circumference, based on standardized pediatric growth charts like World Health Organization (WHO) and Centers for Disease Control and Prevention (CDC) growth standards. It also involves creating features like developmental velocity, calculated as $\Delta D/\Delta t=\left(D\left(t_{2}\right)-\right.$$\left.D\left(t_{1}\right)\right)/\left(t_{2}-t_{1}\right)$, reflecting the rate of change in developmental milestone scores between assessments. Additionally, it covers quantifying cumulative exposure to risk factors, such as days with poor air quality and screen time during early childhood, generating features for adherence to pediatric guidelines like vaccination completeness and well-child visit schedules, and creating interaction terms reflecting synergistic effects in child health, such as genetic predispositions interacting with environmental exposures.

To reduce dimensionality, mitigate the risk of overfitting (especially with potentially limited sample sizes in specific pediatric subpopulations), and improve model training efficiency and interpretability, various feature selection techniques are used. These are broadly categorized into filter methods, wrapper methods, and embedded methods. Filter methods evaluate features independently of the AI model, using statistical measures relevant to pediatric outcomes, such as the chi-square test for assessing associations between categorical risk factors (eg, maternal smoking during pregnancy) and a binary child health outcome, and the ANOVA *F* value for assessing the relationship between numerical predictors (eg, birth weight) and different risk groups. Wrapper methods, such as recursive feature elimination, use the performance of a specific AI model to iteratively build and evaluate models with different subsets of features. Embedded methods perform feature selection as an integral part of the model training process. For instance, tree-based algorithms like random forest and gradient boosting inherently provide feature importance scores based on how much each feature contributes to reducing impurity or error across the ensemble of trees. Regularization techniques, such as L1 regularization (Lasso), are particularly useful. The Lasso objective function for a linear model predicting a child’s health outcome y based on features X and coefficients β is as follows:


(3) $$L_{\text{Lasso}}=\sum_{i=1}^{n}\;{\left(y_{i}-\beta_{0}-\sum_{j=1}^{p}\;\;x_{ij}\beta_{j}\right)}^{2}+\lambda \sum_{j=1}^{p}\;\left|\beta_{j}\right|$$

where λ is the regularization parameter that controls the penalty on the sum of absolute values of the coefficients, effectively shrinking less important feature coefficients to zero. The selection of λ is typically done via cross-validation.

To provide an overview of the preprocessing pipeline and feature engineering strategies across different data modalities, the key steps are summarized in Table 3.

**Table 3.** Summary of pediatric data preprocessing steps and feature engineering strategies.

Data type	Preprocessing methods	Feature engineering	Examples
Growth indicators	Missing data imputation (KNN^a^ and MICE^b^)	*z* score calculation and velocity features	Age- and sex-adjusted *z* scores
Early exposure	Outlier detection (*z* score and IQR)	Cumulative exposure and interaction terms	Poor air quality days and screen time
Questionnaires	Data cleaning and encoding	One-hot encoding and label encoding	Vaccination scores and visit adherence
School records	Data integration	Feature extraction	Immunization records and attendance
Wearables	Noise reduction and smoothing	Activity patterns and sleep metrics	Activity levels and sleep duration
Genomics	Data anonymization	Genetic risk scoring	Genetic predispositions

a KNN: k-nearest neighbors.

b MICE: multivariate imputation by chained equations.

### Risk Stratification Algorithm

The process of risk stratification involves translating the continuous risk probabilities or composite risk scores generated by AI models into distinct, actionable risk categories that are meaningful in a clinical context. This is achieved by defining specific thresholds that convert model outputs into discrete risk strata. The thresholds are determined through a combination of clinical expertise, statistical methods, and decision theory principles, ensuring they are both clinically relevant and statistically valid. $P\left(Risk\;/\;X_{child}\right)$ represents the feature vector for a specific child. For a 4-tier stratification system, the risk categories can be defined as follows:

- Low risk: $P\left(Risk\;/X_{child}\right)<\theta_{1}$
- Moderate risk: $\theta \leq P\left(Risk\;/\;X_{child}\right)<\theta_{2}$
- High risk: $\theta_{2}\leq P\left(Risk\;/\;X_{child}\right)<\theta_{3}$
- Very high risk: $P\left(Risk\;/\;X_{child}\right)\geq \theta_{3}$

The selection of the thresholds $\left(\theta_{1},\theta_{2},\theta_{3}\right)$ is guided by clinical expertise, statistical methods, and decision theory. Pediatric specialists define thresholds based on established clinical guidelines, acceptable risk levels for specific age groups, and the availability and efficacy of preventive interventions. Statistical methods, such as using percentiles of the predicted risk distribution in a well-characterized pediatric reference population, can also be used. For example, thresholds might be set to correspond to the 50th, 80th, and 95th percentiles of this distribution. Decision theory principles can further refine threshold optimization by considering a utility function that balances the costs and benefits associated with true positives, false positives, true negatives, and false negatives. This might involve analyzing metrics like Youden J statistic (J=sensitivity+specificity−1) or finding points on the precision-recall curve that correspond to desired tradeoffs for pediatric screening or intervention programs.

When the system is designed to predict multiple distinct health risks for a child, such as the risk of obesity, developmental delay, or asthma exacerbation, the framework requires a mechanism to present these risks in a consolidated and understandable manner. This can be achieved by either displaying the stratified risk level for each condition independently or by developing a composite pediatric health vulnerability index (CPHVI). The CPHVI is calculated by assigning weights $w_{i}$ to each risk score *S*($R_{i}$) based on factors, such as the clinical severity of risk $R_{i}$, its impact on long-term child development, its prevalence in the target pediatric population, and its responsiveness to an intervention. To translate the CPHVI into clinical practice, we propose a tiered intervention model based on predefined, clinically meaningful thresholds developed in collaboration with pediatric experts. For example, a CPHVI score exceeding a “high risk” threshold could automatically trigger an alert in the EHR system for the primary care physician, along with a recommendation for a direct referral to a relevant specialist. The formula for the CPHVI is as follows:


(4) $$S_{CPHVI}=\frac{\sum_{i=1}^{N}\;\;w_{i}\cdot S\left(R_{i}\right)}{\sum_{i=1}^{N}\;\;w_{i}}$$

The determination of the weights $w_{i}$ is a complex task, often requiring expert consensus or data-driven approaches. Another advanced approach involves using unsupervised learning techniques, such as clustering algorithms, applied to the vector of predicted individual risk probabilities for each child. This can help identify common co-occurring risk patterns or distinct pediatric subphenotypes that share similar multirisk profiles, guiding more tailored multifaceted interventions.

To enhance clinical utility and facilitate shared decision-making with families, the reasons behind a child’s stratification into a particular risk category must be as transparent and interpretable as possible. Techniques, such as SHAP and Local Interpretable Model-Agnostic Explanations (LIME), are used. For a given prediction $f\left(X_{\text{child}}\right)$, SHAP assigns an importance value (SHAP value, $\phi_{j}$) to each input feature j, representing its marginal contribution to pushing the prediction away from a baseline. The sum of SHAP values for all features plus the baseline prediction equals the model’s output for that child:


(5) $$f\left(X_{\text{child }}\right)=\phi_{0}+\sum_{j=1}^{M}\;\phi_{j}$$

This allows clinicians to understand which specific factors (eg, low birth weight, specific dietary patterns, and lack of physical activity) are most influential in determining a child’s assessed risk level, thereby guiding targeted advice and interventions.

### BERT-Based Knowledge Extraction and NLP Modeling

BERT-based NLP is used to extract informative representations from unstructured pediatric clinical text (eg, consultation records, clinical notes, and parental narratives), which are subsequently used as input features for pediatric health risk stratification models.

Pediatric medical journals serve as a vital source of pediatric disease knowledge, encompassing a wealth of information on pediatric diseases, including clinical symptoms, diagnostic methods, treatment approaches, and prognoses. These journals are written by pediatric experts and researchers and are based on extensive clinical practice and scientific research. They contain detailed case studies, research findings, and expert opinions, providing a solid foundation for the extraction and application of pediatric disease knowledge. The knowledge derived from these journals can help health care professionals better understand the characteristics and progression of pediatric diseases, thereby improving the quality of pediatric health care services [14].

The pediatric disease knowledge base is a structured repository that integrates information from pediatric medical journals and other authoritative sources. It includes various aspects of pediatric diseases, such as disease names, symptoms, signs, laboratory test results, imaging findings, treatment methods, and prognoses. This knowledge base is designed to provide comprehensive and accurate information on pediatric diseases, supporting clinical decision-making and research. By organizing and structuring the information from pediatric medical journals, the pediatric disease knowledge base enables efficient retrieval and utilization of pediatric disease knowledge, facilitating the application of this knowledge in clinical practice and research.

The process begins with the ingestion of pediatric medical journal data into our system. These textual data, rich in pediatric disease knowledge, undergo meticulous preprocessing to align with BERT’s input requirements. The text is tokenized into a sequence of tokens $\left[T_{1},T_{2},\ldots ,T_{n}\right]$, where each $T_{i}$ represents a word or subword unit. Additionally, the pediatric disease knowledge base is incorporated to enhance the model’s understanding of pediatric medical concepts. BERT’s architecture consists of multiple transformer encoder layers, each equipped with self-attention mechanisms. The input tokens, enriched with special markers like [CLS] and [SEP], pass through these layers, generating a matrix of contextualized embeddings $z=\left\{z_{1},z_{2},\ldots ,z_{n}\right\}$. These embeddings capture intricate semantic relationships and contextual information within the text, forming the basis for subsequent knowledge extraction tasks.

To identify key pediatric disease entities and their relationships within the text, named entity recognition and relation extraction techniques are applied to the BERT-generated embeddings. For named entity recognition, a linear layer combined with a conditional random field is used. The conditional random field loss function is defined as follows:


(6) $$L_{NER}=-\frac{1}{N}\sum_{i=1}^{N}logP\left(y_{i}|X_{i}\right)$$

where N is the number of samples and $P\left(y_{i}|X_{i}\right)$ is the probability of the true label sequence $y_{i}$ given the input sequence $X_{i}$. For relation extraction between entities, a classifier is trained using a cross-entropy loss function as follows:


(7) $$L_{RE}=-\sum_{i=1}^{M}\;\sum_{j=1}^{C}\;y_{i,j}log{\overset{ˆ}{y}}_{i,j}$$

where M represents the number of entity pairs, C is the number of relation types, and $y_{i,j}$ and ${\overset{ˆ}{y}}_{i,j}$ are the true and predicted probabilities for relation *j* between the entity pair *i*, respectively. The extracted entities and relationships are then integrated into the pediatric disease knowledge base. This knowledge base, enriched with entities and relationships from pediatric medical literature, serves to enhance a medical chatbot’s responses. When users input symptoms, the chatbot leverages this knowledge base to generate accurate and contextually relevant diagnostic suggestions and risk assessments.

**Figure 3.**
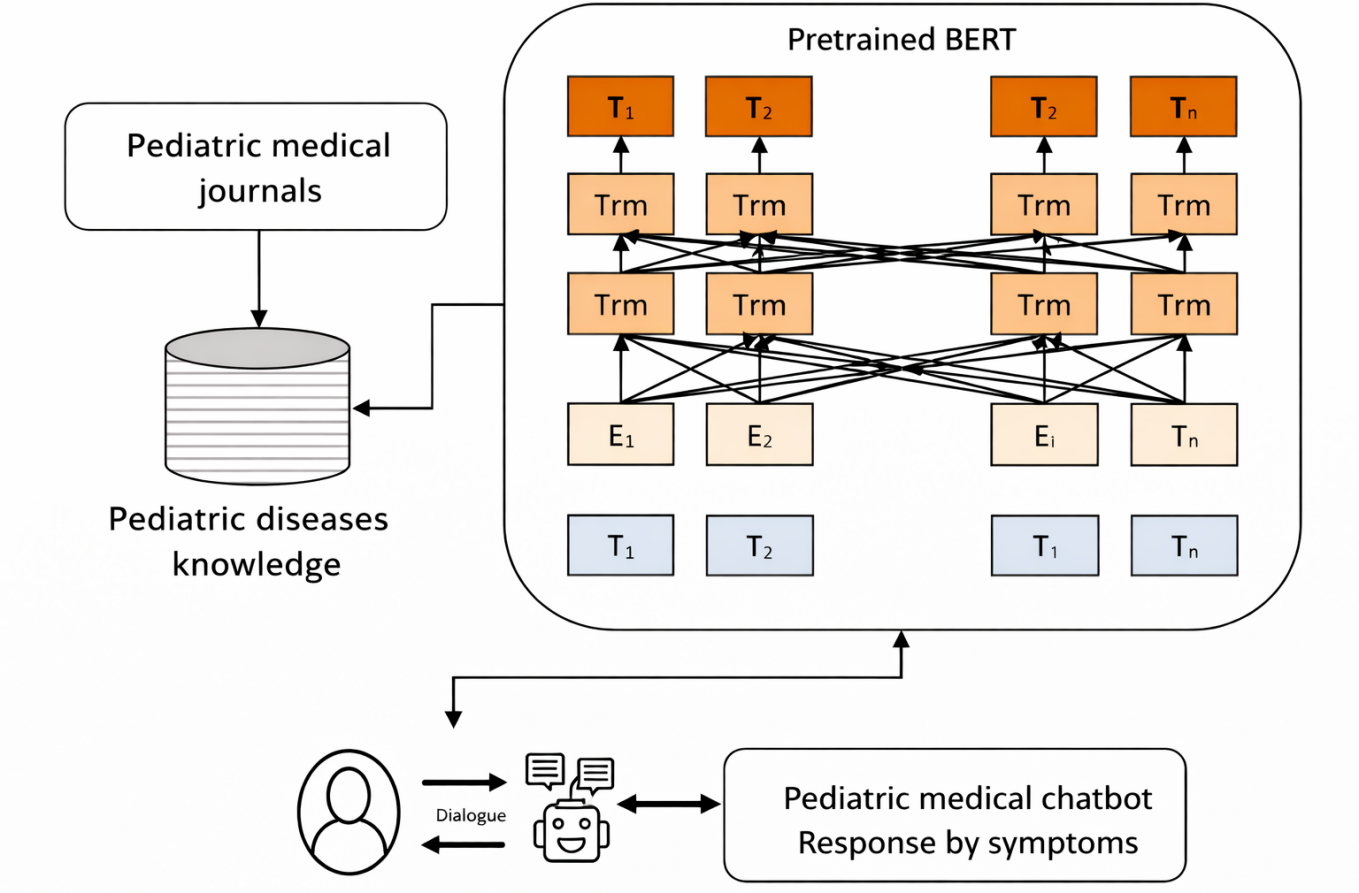
BERT-driven pediatric disease knowledge extraction and medical dialogue response framework. T denotes the input token sequence obtained after tokenization of the pediatric clinical text, E represents the contextualized embedding vectors produced by the BERT encoder, and Trm refers to the Transformer encoder layers of the BERT model. BERT: Bidirectional Encoder Representations From Transformers.

Figure 3 illustrates a comprehensive workflow where BERT’s robust language understanding capabilities are harnessed to extract valuable pediatric disease knowledge from medical texts. This knowledge is subsequently used to empower a medical chatbot, enabling it to deliver precise and informative responses to user inquiries regarding pediatric symptoms. The integration of BERT with pediatric medical journals and a pediatric disease knowledge base provides a powerful tool for advancing pediatric health care through improved diagnostic accuracy and personalized treatment recommendations.

Our knowledge extraction module is built upon BioBERT, a pretrained language model optimized for the biomedical domain. We chose BioBERT as our base model due to its demonstrated strong performance on various biomedical text mining tasks. For fine-tuning, we used a domain-specific corpus comprising over 50,000 articles from leading pediatric medical journals and over 100,000 deidentified clinical notes. The model was fine-tuned for 5 epochs with a learning rate of 2e^−5^. The performance of our fine-tuned model on key NLP tasks was rigorously evaluated on a manually annotated test set of 500 clinical notes, which were dual-annotated by pediatric domain experts to ensure quality.

### System Architecture Design

The successful deployment, scalability, and usability of the AI-driven pediatric health risk stratification framework critically depend on a meticulously planned and well-engineered system architecture. This architecture must robustly support efficient multimodal data processing pipelines; reliable and timely execution of complex AI models; secure and compliant management of sensitive pediatric health data; and an intuitive, actionable interface for diverse end users, including pediatricians, specialist clinicians, public health officials, researchers, and potentially, with appropriate safeguards, parents or caregivers. A multitier, service-oriented architecture is proposed to ensure modularity, maintainability, and scalability. This typically comprises the following tiers:

1. Data tier: This foundational tier is responsible for the persistent storage and comprehensive management of all relevant pediatric health data. This includes raw input data from diverse sources (EHRs, wearables, and questionnaires), preprocessed and feature-engineered datasets, trained AI models (including their versions and metadata), generated risk profiles for individual children, and audit logs. This tier would likely involve a hybrid database strategy, combining relational databases (MySQL) for structured metadata, patient demographics, and well-defined clinical entities, with potentially NoSQL databases for handling large volumes of heterogeneous, unstructured, or streaming data (like continuous sensor readings). Robust data governance, backup, and recovery mechanisms are integral.
2. Application logic tier (backend services): This is the computational core of the system, housing the data preprocessing pipelines, sophisticated feature engineering modules, the AI model inference engine, and the risk stratification algorithms. It handles all computational tasks, business logic for risk assessment, and interactions with the data tier. This tier would be developed using scalable and efficient programming languages and appropriate backend frameworks. It may be implemented as a set of microservices to enhance scalability and independent deployability of different functionalities.
3. Presentation tier (frontend interfaces): This tier provides the user interface and user experience for interacting with the system. This could manifest as a secure web-based application accessible via standard browsers, designed with responsive layouts for use on various devices. For specific user groups like parents or children (with age-appropriate design), a dedicated mobile app might be considered. The frontend allows authorized users to view individualized pediatric risk stratification results, explore interactive visualizations of risk factors and trends, access evidence-based decision support information or guideline recommendations, and potentially (for clinicians) trigger further diagnostic or intervention pathways.

**Figure 4.**
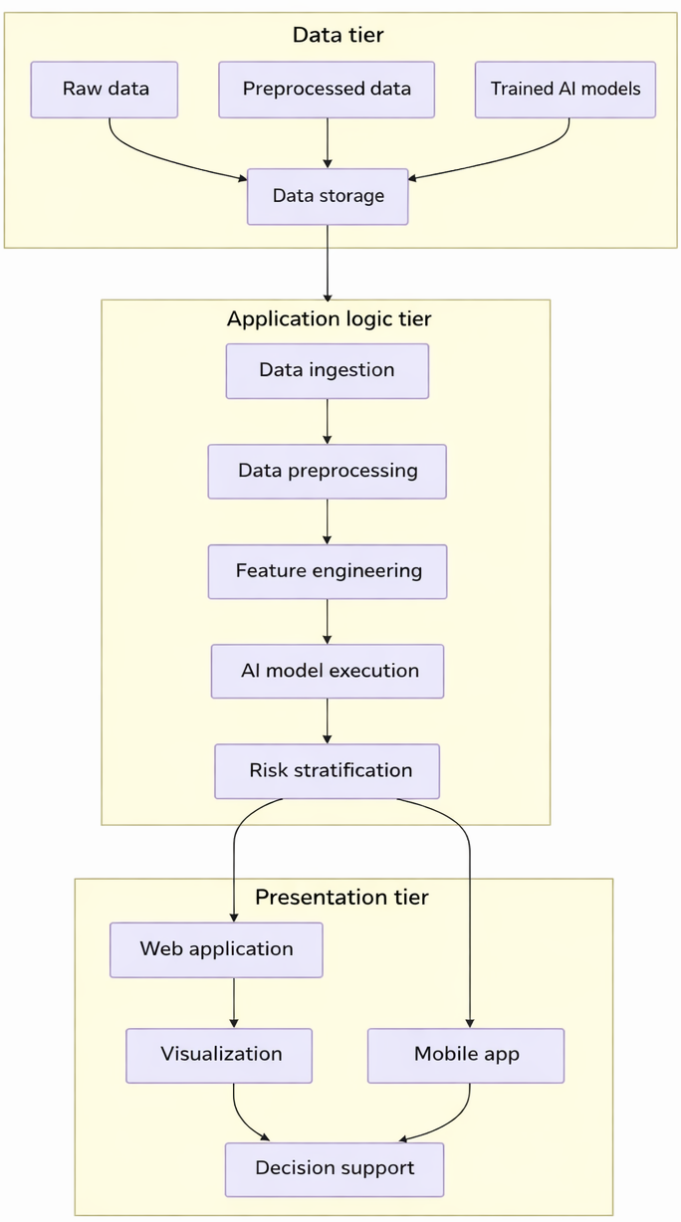
Multitier system architecture diagram for pediatric risk stratification. AI: artificial intelligence.

Key functional system modules are designed to support the end-to-end pediatric risk stratification process (Figure 4).

1. Pediatric data ingestion and management module: It securely handles the import of data from diverse, often disparate, pediatric health information systems and sources. It performs initial data validation and schema mapping and manages data storage, versioning, and provenance tracking, which are crucial for longitudinal pediatric studies.
2. Preprocessing and pediatric feature engineering module: It implements complex algorithms described in the preceding section, tailored for pediatric data. This module needs to be highly configurable and extensible to accommodate new data types, evolving pediatric clinical knowledge, or updated feature definitions.
3. AI model execution and management engine: It loads versioned, trained AI models and performs inference on new or updated pediatric patient data to generate risk predictions. This engine must be optimized for performance and scalability, especially if real-time or near real-time risk assessment is required for acute pediatric conditions. It also manages the model lifecycle, including retraining triggers and performance monitoring.
4. Pediatric risk stratification and profiling module: It implements the sophisticated algorithms from the preceding section to convert raw model outputs into clinically interpretable risk strata and comprehensive, multidimensional pediatric risk profiles, potentially including age-adjusted interpretations.
5. Reporting, visualization, and alerting module: It generates customized reports, interactive dashboards (eg, showing population-level pediatric risk distributions and trends over time for specific age cohorts), and dynamic visualizations (eg, individual child risk timelines and feature importance charts for specific predictions). It may also include an alerting mechanism for clinicians when a child’s risk profile crosses critical, predefined thresholds.
6. User authentication, authorization, and audit module: It ensures secure, role-based access to the system and its sensitive pediatric data, adhering strictly to privacy regulations (eg, HIPAA, GDPR, and Children's Online Privacy Protection Act [COPPA]). Comprehensive audit trails of data access and system actions are maintained.
7. Interoperability layer (application programming interfaces [APIs]): It provides well-defined APIs, possibly using standards like Fast Healthcare Interoperability Resources, to allow secure data exchange and interaction with other clinical systems (eg, pediatric EHRs, laboratory information systems, and clinical decision support tools embedded in existing workflows).

The data flow within the system begins with secure data ingestion from multiple sources into the *data tier*. The *application logic tier* then orchestrates the preprocessing and feature engineering pipelines. The processed features are subsequently fed into the *AI model execution engine* for risk prediction. These predictions are passed to the *risk stratification and profiling module*, and the final, interpretable pediatric risk profiles are stored and made available for secure access and visualization through the *presentation tier* or via the *API layer*. The technology stack will be carefully chosen based on criteria, such as scalability for large pediatric populations, real-time performance requirements, security mandates for child data, ease of development and maintenance, and compatibility with existing health care IT infrastructure. This might include Python with libraries like Pandas, NumPy, Scikit-learn, TensorFlow/Keras, and PyTorch for AI or ML development; web frameworks, such as Django/Flask (Python), Spring Boot (Java), and Node.js, for the backend services; modern JavaScript frameworks like React, Angular, and Vue.js for building responsive and interactive frontend interfaces; and a combination of SQL and NoSQL databases as described for the *data tier*. Containerization technologies like Docker and orchestration tools like Kubernetes may be used for deployment and scaling.

### Prototype System Implementation

**Figure 5.**
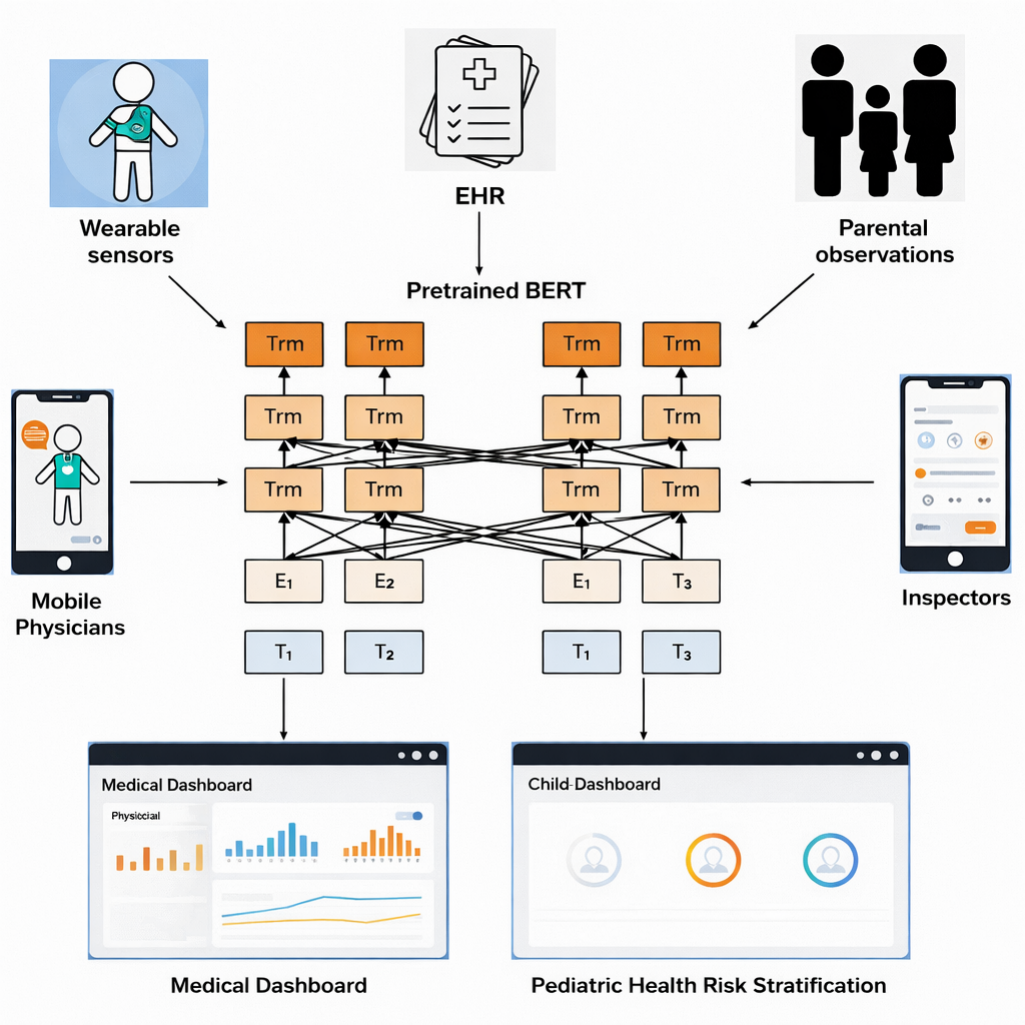
Structure of the pediatric health risk stratification prototype system. T denotes the input token sequence obtained after tokenization of the pediatric clinical text, E represents the contextualized embedding vectors produced by the BERT encoder, and Trm refers to the Transformer encoder layers of the BERT model. BERT: Bidirectional Encoder Representations From Transformers; EHR: electronic health record.

The pediatric health risk stratification prototype system integrates multisource data input with advanced AI models to deliver a comprehensive risk assessment platform. As illustrated in Figure 5, the system collects diverse data types, including patient demographics, clinical records, and parent-reported outcomes, which serve as the foundation for subsequent analysis and risk assessment. This integration ensures a holistic view of pediatric health, enabling more accurate and nuanced risk predictions.

At the core of the system is a pretrained BERT model, specifically fine-tuned to handle pediatric health terminology and context. The model processes input text data through multiple Transformer modules, which encode the text to capture complex linguistic patterns and semantic relationships. Feature extraction layers then distil these encoded representations into risk-relevant features. These features are passed to risk assessment components where the actual risk stratification takes place, transforming raw data into actionable insights regarding children’s health risks.

The system’s implementation leverages a robust technology stack to ensure efficiency, scalability, and user-friendliness. The backend, developed using Python and its rich library ecosystem, handles data processing and model execution. It uses microservices, potentially orchestrated by lightweight frameworks like Flask, to manage distinct functionalities such as data ingestion, preprocessing, and model inference. Data storage follows a hybrid approach, combining PostgreSQL for structured data with MongoDB for unstructured or semistructured content. The frontend, built with modern JavaScript frameworks, offers tailored interfaces for different users, including physicians and parents. It includes features like risk profile visualizations and population-level risk trend dashboards. Docker is used for containerization to ensure smooth deployment and scalability of the application components.

To illustrate the practical utility of the framework, consider a hypothetical clinical scenario. Dr Smith, a pediatrician, begins her day by logging into the system’s dashboard. The system flags one of her patients, a 5-year-old child, for a significant increase in the composite risk score for developing obesity. Dr Smith clicks on the patient’s profile and is presented with an interactive, multimodal dashboard. An explainable AI feature, using SHAP values, highlights the primary contributing factors: a recent decrease in physical activity levels captured by wearable data and parent-reported dietary logs indicating high consumption of processed foods. Based on these insights, the system provides Dr Smith with evidence-based, actionable recommendations, including a referral to a pediatric nutritionist and a set of tailored educational materials for the parents. This scenario demonstrates how the framework can transform raw data into clinically actionable insights to facilitate timely and personalized preventive care.

### Experimental Setup

The primary dataset used for training and validating the AI models included data from over 40,000 pediatric participants, with an age range of 2 to 8 years at the time of data collection or follow-up. The dataset was partitioned into 3 mutually exclusive sets: a training set (70% of the data), a validation set (15%), and an independent holdout test set (15%). To ensure temporal validity if longitudinal data were used, the split was performed such that data from earlier time periods were used for training and validation, while data from later periods were reserved for testing, mimicking a prospective evaluation.

To benchmark the performance of the proposed AI-driven framework, a selection of robust baseline models was implemented and rigorously evaluated on an identical dataset and predictive task. These comparators included established traditional statistical models frequently used in pediatric risk prediction, specifically logistic regression for binary risk outcomes, where the probability of risk P(Y=1∣X) is modeled as $P\left(Y=1|X\right)={\left(1+e^{-\left(\beta_{0}+\sum_{i=1}^{p}\;\;\beta_{i}X_{i}\right)}\right)}^{-1}$, and Cox proportional hazards models in scenarios where time-to-event data for risk onset were available. Furthermore, simpler yet effective ML algorithms, namely support vector machine (SVM), configured with various kernels (linear and radial basis function), and standard single decision trees, were also incorporated as baselines. In instances where existing, validated pediatric risk scores or established rule-based systems were pertinent to the specific health outcomes under investigation and applicable to the dataset, these were also included in the comparative analysis to provide a comprehensive performance context. To rigorously assess the statistical significance of our model’s superior performance, we used the DeLong test to compare the area under the receiver operating characteristic curve (AUC-ROC) of our proposed model with that of each baseline model.

### Ethical Considerations

This study involved research with human participants. The study protocol was reviewed and approved by the Institutional Review Board and Research Ethics Committee of Hunan University of Arts and Science (HUAS-20250504). All procedures were performed in accordance with the ethical standards of the responsible institutional committee and with the principles of the Declaration of Helsinki. Informed consent was obtained from all participants prior to their inclusion in the study. Participants were fully informed about the purpose of the study, the procedures involved, and their right to withdraw at any time without penalty. The privacy and confidentiality of all participants were strictly maintained throughout the study. All data were anonymized prior to analysis, and no personally identifiable information was collected, stored, or reported. No financial or material compensation was provided to participants for their participation in this study.

## Results

This section reports the empirical performance of the proposed AI-driven pediatric health risk stratification framework evaluated on the independent test set, in comparison with multiple baseline models.

Table 4 summarizes the predictive performance of the proposed model and baseline methods across targeted pediatric health risks. The proposed model achieved an AUC-ROC of 0.85 (95% CI 0.82‐0.88), an area under the precision-recall curve of 0.70 (95% CI 0.65‐0.75), a sensitivity of 0.78, a specificity of 0.80, and an *F*_1_-score of 0.75. In comparison, logistic regression, SVM, random forest, gradient boosting, and a conventional deep learning model yielded lower AUC-ROC and *F*_1_-score values.

**Table 4.** Key performance metrics of the proposed artificial intelligence model and baseline models for targeted pediatric risks in the test set.

Model name	AUC-ROC^a^, value (95% CI)	AUC-PR^b^, value (95% CI)	Sensitivity	Specificity	*F*_1_-score	Brier score
Proposed BERT^c^ model	0.85 (0.82‐0.88)	0.70 (0.65‐0.75)	0.78	0.80	0.75	0.15
Logistic regression	0.72 (0.68‐0.76)	0.60 (0.55‐0.65)	0.65	0.70	0.62	0.20
SVM^d^	0.75 (0.71‐0.79)	0.65 (0.60‐0.70)	0.70	0.72	0.66	0.18
Random forest	0.78 (0.74‐0.82)	0.67 (0.62‐0.72)	0.72	0.75	0.69	0.17
Gradient boosting	0.80 (0.77‐0.83)	0.69 (0.64‐0.74)	0.75	0.76	0.72	0.16
Deep learning	0.83 (0.80‐0.86)	0.72 (0.67‐0.77)	0.76	0.79	0.74	0.14

a AUC-ROC: area under the receiver operating characteristic curve.

b AUC-PR: area under the precision-recall curve.

c BERT: Bidirectional Encoder Representations From Transformers.

d SVM: support vector machine.

The DeLong test demonstrated that the AUC-ROC of the proposed model was significantly higher than that of each baseline model (all *P*<.05). Paired *t* tests on *F*_1_-scores similarly indicated statistically significant improvements in predictive accuracy for the proposed model relative to all comparators.

To further assess alignment with expert judgment, a manual comparison evaluation was conducted using 100 randomly selected pairs of online consultation cases. Automated predictions and expert assessments were concordant in 78 cases, corresponding to an agreement rate of 78%.

**Figure 6.**
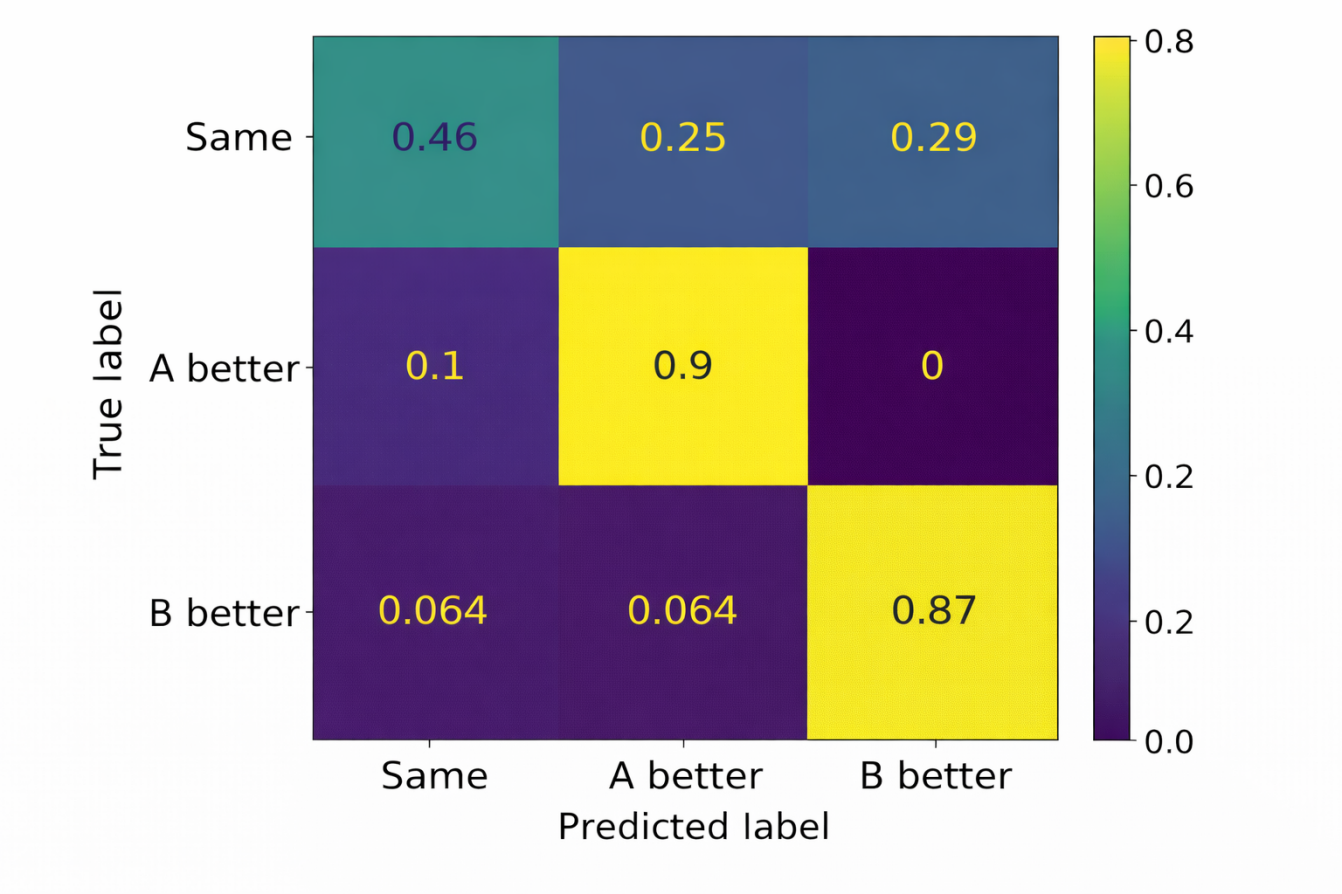
Artificial intelligence prediction confusion matrix.

A confusion matrix (Figure 6) shows that most discrepancies occurred in cases manually labeled as “equivalent,” whereas predictions for clearly differentiated cases exhibited higher agreement with expert assessments. Misclassifications were predominantly observed within the equivalent category, while the majority of nonequivalent cases were correctly identified.

Model performance was further examined across predefined pediatric subgroups, including age categories, sex, and socioeconomic status (Table 5). Comparable AUC-ROC and *F*_1_-score values were observed across all subgroups. For age-based stratification, the AUC-ROC values were 0.84 for children younger than 2 years, 0.85 for those aged 2‐5 years, and 0.86 for those older than 5 years. Similar performance consistency was observed across sex and socioeconomic strata.

**Table 5.** Model performance across different pediatric subgroups for early childhood obesity.

Group	AUC-ROC^a^, value (95% CI)	*F*_1_-score
Age		
<2 years	0.84 (0.80‐0.88)	0.74
2‐5 years	0.85 (0.82‐0.89)	0.75
>5 years	0.86 (0.83‐0.89)	0.76
Sex		
Male	0.85 (0.82‐0.88)	0.75
Female	0.84 (0.81‐0.87)	0.74
Status		
Low SES^b^	0.83 (0.79‐0.87)	0.73
Medium SES	0.85 (0.82‐0.88)	0.75
High SES	0.86 (0.83‐0.89)	0.76

a AUC-ROC: area under the receiver operating characteristic curve.

b SES: socioeconomic status.

## Discussion

### Conclusions

This study successfully designed and validated a novel AI-driven framework for comprehensive pediatric health risk stratification. The primary findings were threefold. First, the framework demonstrates the capability to effectively integrate heterogeneous, multimodal data sources to create a holistic view of a child’s health status. Second, our proposed predictive model, which combines a fine-tuned BERT architecture with an ensemble learning strategy, significantly outperformed established baseline models, such as logistic regression and SVM, in predicting key health risks, achieving an AUC-ROC of 0.85 for early childhood obesity. Third, the prototype system’s risk assessments showed substantial agreement with manual expert evaluations (78% accuracy), confirming its potential clinical utility and feasibility. The successful implementation of the prototype system, featuring intuitive dashboards for both clinicians and parents, further illustrates the practical applicability of this approach in facilitating early and personalized interventions, thereby contributing a novel and robust technological foundation for proactive pediatric health care.

### Limitations and Future Work

While the presented framework shows considerable promise, certain limitations and avenues for future research warrant discussion. The current validation, though rigorous, was based on a specific dataset of over 40,000 pediatric participants aged 2‐8 years. Broader validation across more diverse pediatric populations and longitudinal follow-up are essential to ascertain long-term predictive accuracy and generalizability. One limitation of our study is the observed slight dip in model performance for the subgroup of children aged 3‐5 years, which we attribute to the relative scarcity of rich, unstructured text data for this age cohort. To mitigate this issue in future work, we plan to incorporate alternative text sources. A promising approach, as suggested, is the inclusion of open-ended responses from parental questionnaires. These narratives, which capture detailed parental concerns and observations, can be processed by our fine-tuned BERT model to generate rich semantic features, thereby enriching the feature set for younger children and addressing the data sparsity issue. A crucial direction for future work is the implementation of advanced bias mitigation techniques. We plan to further explore methods, such as adversarial debiasing, which involves training the model to make predictions that are invariant to sensitive attributes, thereby proactively enhancing the fairness and equity of our risk stratification framework.

## Abbreviations

AI: artificial intelligence
API: application programming interface
AUC-ROC: area under the receiver operating characteristic curve
BERT: Bidirectional Encoder Representations From Transformers
CPHVI: composite pediatric health vulnerability index
EHR: electronic health record
GDPR: General Data Protection Regulation
HIPAA: Health Insurance Portability and Accountability Act
MICE: multivariate imputation by chained equations
ML: machine learning
NLP: natural language processing
SHAP: Shapley additive explanations
SVM: support vector machine
