# Correction: Comprehensive Pediatric Health Risk Stratification Using an AI-Driven Framework in Children Aged 2 to 8 Years: Design and Validation Study

**DOI:** 10.2196/92491

**Published:** 2026-02-27

**Authors:** Jundan Chen, Zhihe Mao

**Affiliations:** 1 School of Physical Education, Hunan University of Arts and Science, 3150 Dongting Road, Changde, Hunan, 415000, China, 86 13789072016, 86 07367283047

In “Comprehensive Pediatric Health Risk Stratification Using an AI-Driven Framework in Children Aged 2 to 8 Years: Design and Validation Study” [1], the authors noted two errors.

The author list has been corrected from the following:

Zhihe Mao, PhD; Jundan Chen, PhD

The author list now reads:

Jundan Chen, PhD; Zhihe Mao, BA

Author ZM’s contact information has been corrected to the following:

Phone: 86 13789072016

Fax: 86 07367283047

The correction will appear in the online version of the paper on the JMIR Publications website, together with the publication of this correction notice. Because this was made after submission to PubMed, PubMed Central, and other full-text repositories, the corrected article has also been resubmitted to those repositories.
